# Updates and Current Perspectives of Psychiatric Assessments after Traumatic Brain Injury: A Systematic Review

**DOI:** 10.3389/fpsyt.2016.00095

**Published:** 2016-06-14

**Authors:** Ana Luiza Zaninotto, Jessica Elias Vicentini, Felipe Fregni, Priscila Aparecida Rodrigues, Cibele Botelho, Mara Cristina Souza de Lucia, Wellingson Silva Paiva

**Affiliations:** ^1^Laboratory of Neuromodulation, Center for Clinical Research Learning, Harvard Medical School (HMS), Charlestown, MA, USA; ^2^Department of Neurology, School of Medicine, University São Paulo (USP-SP), São Paulo, Brazil; ^3^Department of Neurology, School of Medical Sciences, State University of Campinas (UNICAMP), Campinas, Brazil; ^4^Division of Psychology, School of Medicine, University São Paulo (USP-SP), São Paulo, Brazil

**Keywords:** traumatic brain injury, psychiatry, depression, anxiety, posttraumatic stress disorder, assessment, scales, inventory reporting, questionnaires

## Abstract

Neuropsychological and psychiatric disorders represent a major concern and cause of disabilities after the trauma, contributing to worse recovery after traumatic brain injury (TBI). However, the lack of well-defined parameters to evaluate patient’s psychiatric disorders leads to a wide range of diagnoses and symptoms. The aim of this study was to perform a review of literature in order to gather data of the most common scales and inventories used to assess and diagnose depression, anxiety, and posttraumatic stress disorder (PTSD) after TBI. We conducted a literature search *via* MEDLINE, PubMed, and Web of Science. We included reviews, systematic reviews, and meta-analysis studies, and we used the following keywords: “traumatic brain injury OR TBI,” “depression OR depressive disorder,” “anxiety,” and “posttraumatic stress disorder OR PTSD.” From 610 titles, a total of 68 systematic reviews or meta-analysis were included in the section “Results” of this review: depression (*n* = 32), anxiety (*n* = 9), and PTSD (*n* = 27). Depression after TBI is a more established condition, with more homogeneous studies. Anxiety and PTSD disorders have been studied in a heterogeneous way, usually as comorbidity with other psychiatric disorders. Some scales and inventories designed for the general community may not be appropriate for patients with TBI.

## Introduction

Globally, traumatic injuries are responsible for more than five million deaths annually, and traumatic brain injury (TBI) is one of the leading causes of disabilities and death. It is estimated that 1.7 million cases of TBI occur each year in the United States (USA), resulting in 52,000 deaths (1). Therefore, TBI represents around one-third (30.5%) of all injury-related deaths in the USA (1).

Traumatic brain injury usually results in brain disorders, leading to a heterogeneous spectrum of morbidities, ranging from transitory disturbances to permanent symptoms (2–6). Cognitive and psychiatric disorders are the common causes of disabilities and may cause difficulties in recovery after TBI (7–10). In diffuse axonal injury, disruption of the neural circuitry between the prefrontal cortex and limbic system (11, 12) can result in mood disorders arising even weeks or months after the initial injury (13).

Gordon et al. (14) reviewed rehabilitation in TBI and highlighted the need for a better understanding of the dynamics of recovery. They argue that only a few studies used measures accepted as “gold standards” (14). In 2010, the National Institute of Neurological Disorders and Stroke (NINDS) Common Data Elements (CDE) was created to develop data standards for clinical research in patients with TBI (15). However, even with the proposal of some guidelines, psychiatric functions are still being assessed in a heterogeneous manner (16, 17). Therefore, the aim of this study is to summarize the literature, including reviews, systematic reviews, and meta-analyses, regarding the scales and inventories most commonly used to diagnose and evaluate depression, anxiety, and posttraumatic stress disorder (PTSD) in patients with TBI. To assess a large number of published articles, we used an original method in order to have a global view of the instruments used in diagnosis over the years.

## Methods

We conducted a literature search *via* online databases including MEDLINE, PubMed, and Web of Science. We included reviews, systematic reviews, and meta-analysis studies. In our search, we used the following keywords: “traumatic brain injury OR TBI,” “depression OR depressive disorder,” “anxiety,” and “posttraumatic stress disorder OR PTSD.” Abstracts and full text were carefully read, and studies were included in our review if they fulfilled the following inclusion criteria: (a) description/citation of the scale, questionnaire, or inventory used, (b) published in a peer-reviewed journal, (c) description of quantitative assessment for diagnosis, (d) full text written in English, and (e) adult participants. We selected studies published up to February 2016.

Searching and data analysis were performed by Ana Luiza Zaninotto and Jessica Elias Vicentini, both of whom have experience with mental health intervention and clinical research in TBI. All reviews and full text were read by the two reviewers and were included if they met the above-mentioned inclusion criteria. This selection method follows previous literature (18).

## Results

We reviewed 610 titles and abstracts and selected studies according to our inclusion and exclusion criteria. Of those, 362 studies were excluded, of which 248 were reviewed entirely (full text). Sixty-nine studies were included in the review focusing on one or more aspects of the following three topics of interest: (a) depression (*n* = 32), (b) anxiety (*n* = 9), and (c) PTSD (*n* = 27). Of the 68 studies, 11 studies had overlapping topics of interest, since they met criteria for more than one psychiatric disorder (17–27). The abstracts and full text that did not meet the inclusion criteria were excluded from the review (*n* = 541). The main reasons for exclusion were that the studies did not report the instruments used to assess the psychiatric disorders and/or the psychiatric assessment was not the center of the study (*n* = 447). The remaining excluded articles (*n* = 94) were either not related to TBI samples, focused on the neurological basis of the psychiatric disease, discussed pharmacological interventions, or did not focus on psychiatric disorders (depression, anxiety, or PTSD).

**Figure 1:**
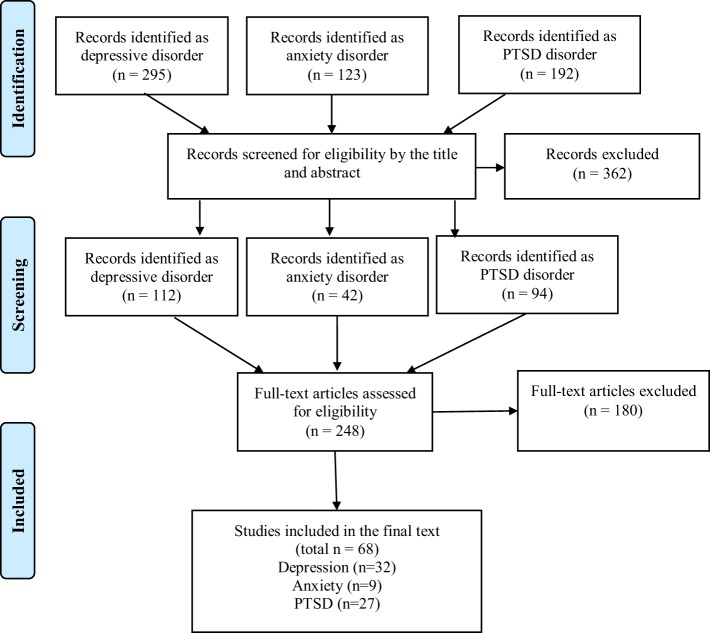
**Flow diagram**.

Each step of the search and review process is detailed in a flow diagram (Figure 1), based on the PRISMA work group (28).

In Table 1, we present studies (*n* = 31) that assessed depressive symptoms or diagnosed depression following TBI. Five of these were meta-analyses. Seven studies analyzed assessments of depression related to TBI in veterans, military personnel, or war-related injuries. We observed that the Beck Depression Inventory (BDI) was presented in all the studies, followed by Structured Clinical Interview for DSM (SCID) and Diagnostic and Statistical Manual (DSM) diagnosis criteria. For TBI populations, the BDI, Symptoms Checklist (SCL), and Center for Epidemiologic Studies-Depression Scale (CES-D) were the most cited self-reported scales. For diagnosis of depression, DSM criteria were the most commonly used, followed by International Classification of Diseases (ICD). The most commonly used instrument was the Hamilton Depression Rating Scale (HAM-D). Table 2 shows different instruments were cited to assess depression.

**Table 1:** **Characteristics of the studies and the scales and inventories used to assess depressive disorder after TBI**.

Reference	Study design	Study population	Number of analyzed studies/total of studies	Instruments
Adamson et al. (29)	Meta-analysis	Neurologic patients	(3/26)	BDI, HADS
Barker-Collo et al. (30)	Meta-analysis	TBI	13	BDI, BDI-II, CES-D, HAM-D, LSSAD, SCL-90-R
Capehart and Bass (19)	Review	Veterans with TBI and PTSD	N/A	HAM-D, BDI
Cooper et al. (31)	Systematic review	Military veterans with mTBI	(4/19)	BDI, BDI-II
Crisp (32)	Systematic review	MDD, SCI, TBI, CBP, MI/CG	(8/54)	BDI, GHQ, CES-D, HAM-D, SCL-90-R
Daggett et al. (33)	Systematic review	Combat veterans who had sustained TBI	(3/17)	BDI, CES-D, CPRS, SCID, VAS-D
Fann et al. (34)	Systematic review	TBI	26	BDI, BDI-II, BPRS, DSM-III-R, HADS, HAM-D, MADRS, PHQ-9, SCL-90-R
Fleminger et al. (35)	Review	TBI	(9/N/A)	BDI, DSM-IV, HAM-D, NFI, SCL-R-90
Garrelfs et al. (20)	Systematic review	ABI	(6/7)	BDI, HADS, HAM-D, SCID-I
Gordon et al. (14)	Systematic review	TBI	N/A	BDI, BDI-II, DSM-IV, CES-D, MCMI, MMPI II, NFI SCL-90-R, SCID
Halbauer et al. (36)	Review	War-related mild to moderate TBI	N/A	BDI, DSM-III, DSM-III-R, DSM-IV-TR, NFI
Hesdorffer et al. (37)	Systematic review	TBI	(4/N/A)	ICD-9-CM; DIS; PSE, SCID
Kim et al. (21)	Systematic review	TBI	(15/66)	BDI, BDI-II, SCID, DSM III, DSM-III-R, DSM-IV, CES-D, NFI, NIMH-DIS, mNIMH-DIS, SCID-I, Wimbledon-SRS
Matarazzo et al. (18)	Systematic review	Veterans with TBI	3	BDI-II, SCID
Menzel (38)	Systematic review	TBI in elderly	1	GDS
Nowrangi et al. (39)	Review	TBI with suicidal risk	N/A	BDI, HAM-D, SCID
O’Neil et al. (22)	Systematic review	Veterans/military population with mTBI	(8/31)	BDI-II, DSM-IV, HADS, SCID-I
O’Neil et al. (23)	Systematic review	Veterans/military population with mTBI	(8/31)	BDI-II, DSM-IV, HADS, SCID-I
Osborn et al. (17)	Meta-analysis	Closed TBI	93	BDI-II, BDICES-D, CIDI, CIS, DIS, DSM-III, DSM-IV, GDS, HADS, ICD-10, LSSAD, MADRS, MINI, SCAN, SCID, PSE, PHQ-9, NFI, SADS-L, SCID, ZSDS
Oyesanya and Ward (40)	Systematic review	Woman with TBI	12	BDI-II, Adaptation of BRFSS, CES-D, HADS, DSM-IV
Panayiotou et al. (24)	Meta-analysis	mTBI	(9/11)	BDI, CES-D, HAM-D, NBAP, POMS, SCL-90, SCL-90-R, ZSDS
Rogers and Read (25)	Systematic review	TBI	(13/N/A)	CES-D, CID, CID-9-CM, DSM-III, DSM-IV, DIS, HAM-D, MMPI, NFI, PSE, PTSD-I, SCAN, SCL-90-R
Rosenthal et al. (41)	Systematic review	TBI	30	BDI, BPRS, CAQ, DSM-III, DSM-III-R, LSSAD, HSCL, HAM-D, MMPI, NIMH-DIS, PACL, PAI, PSE, POMS, SCL-90-R, ZSDS
Sherer et al. (42)	Systematic review	TBI	23	BDI, HADS, NEO-PI-R, Wimbledon-SRS
Simpson and Tate (43)	Review	TBI	19	BDI, BHS, DSM-III-R, PSE
Soo and Tate (26)	Systematic review	mTBI	3	BDI, SCL-90-R
Stalder-Luthy et al. (44)	Meta-analysis	ABI	13	BDI, BDI-II, CIQ, CES-D, CSA, DASS-21, DDS, ERS, GAS, GSI, HADS, MHLC, POMS, PSS, PHQ-9, RSE, SCL-90, SIP, TSK
Steel et al. (45)	Systematic review	Traumatic injury	N/A	BDI, BSI-18, CES-D, DISCs, HADS, HAM-D, HSCL-20, NFI, PHQ-9, SCID, ZSDS
Vahle et al. (46)	Review	People with disabilities	(7/N/A)	BDI, BSI, CES-D, DACL, MEDS, TBDI, ZSDS
van Velzen et al. (47)	Systematic review	Traumatic and non-traumatic ABI	22	BDI, NFI, SCID
Warden et al. (27)	Systematic review	TBI	(7/14)	BDI, HAM-D, DSM-IV

*ABI, acquired brain injury; BDI, Beck Depression Inventory 2nd edition; BDI, Beck Depression Inventory; BHS, Beck Hopelessness Scale; BPRS, Brief Psychiatric Ranting Scale; BRFSS, Behavioral Risk Factor Surveillance System; BSI, Brief Symptom Inventory; BSI-18, Brief Symptom Inventory-18; CAQ, Clinical Analysis Questionnaire; CBP, chronic back pain; CES-D, Center for epidemiological studies; CIDI, Composite International Diagnostic Interview; CIS, Clinical Interview Schedule; CPRS, Compreensive Psychopathological Rating Scale; DACL, Depression Adjective Checklist; DASS-21, Depression Anxiety Stress Scale, DIS, Diagnostic Interview Scale; DISCs, Depression Intensity Scale Circles; DSM-III, Diagnostic and Statistical Manual 3rd edition; DSM-IV, Diagnostic and Statistical Manual 4th edition; GDS, Geriatric Depression Scale; GHQ, General Health Questionnaire; HADS, Hospital Anxiety and Depression Scale; HAM-D, Hamilton Depression Rating Scale; HSCL, Hopkins Symptom Checklist; HSCL-20, Hopkins Symptom Checklist-20; ICD- 10, International Classification of Diseases 10th revision; ICD-9, International Classification of Diseases 9th revision; LSSAD, Leeds Scale for the self-assessment of Anxiety and Depression; MADRS, Montgomery–Asberd Depression Rating Scale; MCMI, Millon Clinical Multiaxial Inventory; MDD, Major depressive disorder; MEDS, Medical-Based Emotional Distress Scale; MINI, Mini-International Neuropsychiatric Interview; MI/CG, myocardial infarction/coronary bypass grafting; MMPI, Minnesota Multiphasic Personality Inventory; MMPI-2, Minnesota Multiphasic Personality Inventory 2; mNIMH-DIS, Modified NIMH’s Diagnostic Interview Schedule; mTBI, mild traumatic brain injury; NEO-PI-R, NEO Personality Inventory Revised; NFI, Neurobehavioral Functioning Inventory; NIMH-DIS, NIMH’s Diagnostic Interview Schedule; PACL, Personality Adjective Checklist; PAI, Portland Adaptability Inventory; PHQ-9, Patient Health Questionnaire-9; POMS, Profile of Mood State; PSE, Present State Examination; SADS-L, Schedule for Affective Disorders and Schizophrenia (lifetime); SCAN, Schedules Clinical Assessment in Neuropsychiatry; SCI, spinal cord injury; SCID, Structured Clinical Interview for DSM; SCL 90-R, Symptoms Checklist 90-Revised; STAI, State-Trait Anxiety Inventory; TBDI, Talbieh Brief Distress Inventory; VAS-D, Visual Analog Scale for Depression; Wimbledon-SRS, Wimbledon Self-Report Scale; ZSDS, Zung Self-Rating Depression Scale*.

**Table 2:** **Scales and inventories used to assess depressive disorder after TBI**.

Name of the scale	Abbreviation	Clinical utility
Beck Depression Inventory (48)	BDI	Interview schedule
Beck Depression Inventory 2nd edition (49)	BDI-II	Interview schedule
Beck Hopelessness Scale (50)	BHS	Interview schedule
Behavioral Risk Factor Surveillance System (51)	BRFSS	Interview schedule
Brief Psychiatric Rating Scale (52)	BPRS	Interview schedule
Brief Symptom Inventory (53)	BSI	Interview schedule
Brief Symptom Inventory-18 (54)	BSI-18	Interview schedule
Center for Epidemiologic Studies – Depression form (55)	CES-D	Interview schedule
Clinical Analysis Questionnaire (56)	CAQ	Interview schedule
Clinical Interview Schedule (57)	CIS	Interview schedule
Composite International Diagnostic Interview (58)	CIDI	Interview schedule
Comprehensive Psychopathological Rating Scale (59)	CPRS	Interview schedule
Depression Adjective Checklist (60)	DACL	Interview schedule
Depression Anxiety Stress Scale 21 (61)	DASS-21	Interview schedule
Depression Intensity Scale Circles (62)	DISCs	Interview schedule
Diagnostic and Statistical Manual 3rd edition [DSM-III (63)]	DSM-III	Diagnose
Diagnostic and Statistical Manual 4th edition [DSM-IV (64)]	DSM-IV	Diagnose
Diagnostic Interview Scale (65)	DIS	Interview schedule
General Health Questionnaire (66)	GHQ	Interview schedule
Geriatric Depression Scale (67)	GDS	Interview schedule
Hamilton Depression Rating Scale (68)	HAM-D	Diagnose
Hopkins Symptom Checklist (69)	HSCL	Interview schedule
Hopkins Symptom Checklist-20	HSCL-20	Interview schedule
Hospital Anxiety and Depression Scale (70)	HADS	Interview schedule
International Classification of Diseases 9th revision (71)	ICD-9	Diagnose
International Classification of Diseases 10th revision (72)	ICD-10	Diagnose
Leeds Scale for the self-assessment of Anxiety and Depression (73)	LSSAD	Interview schedule
Mayo-Portland Adaptability Inventory (74)	MPAI	Interview schedule
Medical-Based Emotional Distress Scale (75)	MEDS	Interview schedule
Millon Clinical Multiaxial Inventory (76)	MCMI	Interview schedule
Mini-International Neuropsychiatric Interview (77)	MINI	Interview schedule
Minnesota Multiphasic Personality Inventory – 2 (78)	MMPI-2	Interview schedule
Minnesota Multiphasic Personality Inventory (79)	MMPI	Interview schedule
Montgomery–Asberg Depression Rating Scale (80)	MADRS	Diagnose
NEO Personality Inventory Revised (81)	NEO-PI-R	Interview schedule
Neurobehavioral Functioning Inventory (82)	NFI	Interview schedule
NIMH’s Diagnostic Interview Schedule (83)	NIMH-DIS	Diagnose
NIMH’s Diagnostic Interview Schedule modified (84)	mNIMH-DIS	Diagnose
Patient Health Questionnaire-9 (85)	PHQ-9	Interview schedule
Personality Adjective Checklist (86)	PACL	Interview schedule
Present State Examination (87)	PSE	Interview schedule
Profile of Mood State (88)	POMS	Interview schedule
Schedule for Affective Disorders and Schizophrenia (lifetime) (89)	SADS-L	Interview schedule
Schedules Clinical Assessment in Neuropsychiatry (90)	SCAN	Interview schedule
State-Trait Anxiety Inventory (91)	STAI	Interview schedule
Structured Clinical Interview (92)	SCID	Diagnose
Symptoms Checklist 90-Revised (93)	SCL 90-R	Interview schedule
Talbieh Brief Distress Inventory (94)	TBDI	Interview schedule
Visual Analog Scale for Depression (95, 96)	VAS-D	Interview schedule
Wimbledon Self-Report Scale (97)	Wimbledon-SRS	Interview schedule
Zung Self-Rating Depression Scale (98)	ZSDS	Diagnose

Table 3 shows the nine studies that assessed anxiety disorders after TBI. Eight of these studies overlapped with other psychiatric conditions. Just one review focused on the anxiety sequelae after TBI (99). The Hospital Anxiety and Depressive Symptoms Scale (HADS) was the most cited instrument to assess anxiety, followed by State-Trait Anxiety Inventory (STAI). The DSM criteria were most commonly used to diagnose anxiety. Table 4 shows the instruments cited in the anxiety reviews that were analyzed.

**Table 3:** **Characteristics of the studies and the scales and inventories used to assess anxiety disorder after TBI**.

Reference	Study design	Study population	Number of analyzed studies/total of studies	Instruments
Garrelfs et al. (20)	Systematic review	ABI (TBI = 4)	(3/7)	HADS, HAM-A, NRS, STAI
Moore et al. (99)	Review	mTBI	N/A	BAI, MMPI, MCMI-III, STAI
O’Neil et al. (22)	Systematic review	Veterans/military with mTBI	(6/31)	HADS, NSI
O’Neil et al. (23)	Systematic review	Veterans/military with mTBI	(6/31)	HADS, NSI
Osborn et al. (17)	Meta-analysis	Closed TBI	41	BAI, HADS, DSM-IV; DSM-III-R, DSM-IV; ICD-10, LSSAD, MINI, SCID-I, SCAN, SADS-L; STAI
Panayiotou et al. (24)	Meta-analysis	mTBI	(5/11)	BAI, CAPS, GHQ, HTQ, IES, POMS, SCL-90-R
Rogers and Read (25)	Review	TBI	N/A	BEC, BSQ, DES, DIS, DSM-III, MMPI, PCSSC, DSM-IV, SCAN, SCL-90-R
Soo and Tate (26)	Systematic review	mTBI	3	BAI, IES, SCL-90-R, STAI-S
Warden et al. (27)	Systematic review	TBI	(1/14)	Y-BOCS

*ABI, acquired brain injury; mTBI, mild traumatic brain injury*.

*Scales: BAI, Beck Anxiety Inventory; BEC, Behavior Evaluation Checklist; BSQ, Body Sensations Questionnaire; DES, Dissociative Experience Scale; DIS, Diagnostic Interview Schedule; DSM-III, Diagnostic and Statistic Manual 3rd edition; DSM-IV, Diagnostic and Statistic Manual 4th edition; GHQ, General Health Questionnaire; HADS, Hospital Anxiety and Depression Scale; HAM-A, Hamilton Anxiety Scale; HTQ, Harvard Trauma Questionnaire; ICD-10, International Classification of Diseases; IES, Impact of Events Scale; LSSAD, Leeds Scale for the Self-Assessment of Anxiety and Depression; MCMI-III, Millon Clinical Multiaxial Inventory 3rd edition; MINI, Mini-International Neuropsychiatric Interview; MMPI, Minnesota Multiphasic Personality Inventory; NRS, Neurobehavioral Rating Scale; NSI, Neurobehavioral Symptom Inventory; PCSSC, Post-Concussion Syndrome Symptom Checklist; POMS, Profile of Mood State; PTSD-I, PTSD Interview; SADS, Schedule for Affective Disorders and Schizophrenia; SCAN, Schedules for Clinical Assessment in Neuropsychiatry; SCID-I, Structured Clinical Interview; SCL-90R, Symptoms Checklist, 90R; STAI, State-Trait Anxiety Inventory; Y-BOCS, Yale–Brown Obsessive Compulsive Scale*.

**Table 4:** **Scales and inventories used to assess anxiety disorder after TBI**.

Name of the scale	Abbreviation	Clinical utility
Beck Anxiety Inventory (50)	BAI	Interview schedule
Diagnostic Interview Schedule (65)	DIS	Interview schedule
Diagnostic and Statistic Manual 3rd edition [DSM-III (63)]	DSM-III	Diagnose
Diagnostic and Statistic Manual 3rd edition-revised [DSM-III-R (100)]	DSM-III-R	Diagnose
Diagnostic and Statistic Manual 4th edition [DSM-IV (64)]	DSM-IV	Diagnose
General Health Questionnaire (66)	GHQ	Interview schedule
Hamilton Anxiety Scale (68)	HAM-A	Diagnose
Hospital Anxiety and Depression Scale (70)	HADS	Interview schedule
Impact of Events Scale (101)	IES	Interview schedule
International Classification of Disease (102)	ICD-10	Diagnose
Leeds Scale for the Self-Assessment of Anxiety and Depression (103)	LSSAD	Interview schedule
Millon Clinical Multiaxial Inventory 3rd edition (76)	MCMI-III	Interview schedule
Mini-International Neuropsychiatric Interview (77)	MINI	Interview schedule
Minnesota Multiphasic Personality Inventory (79)	MMPI	Interview schedule
Neurobehavioral Rating Scale-Revised (104)	NRS-R	Interview schedule
Profile of Mood State (88)	POMS	Interview schedule
Schedule for Affective Disorders and Schizophrenia (89)	SADS	Interview schedule
Schedules for Clinical Assessment in Neuropsychiatry (90)	SCAN	Interview schedule
State-Trait Anxiety Inventory (91)	STAI	Interview schedule
Structured Clinical Interview (105)	SCID-I	Diagnose
Symptoms Checklist – 90R (106)	SCL-90R	Interview schedule
Yale–Brown Obsessive Compulsive Scale (107)	Y-BOCS	Interview schedule

In our search, we found 26 reviews and meta-analyses related to PTSD and TBI. We identified two types of studies, one focusing on military veterans or war-related TBI (*n* = 13), and another focusing on a non-specific TBI population (*n* = 13) (Table 5). Table 6 shows a summary of the scales and inventories used to assess PTSD in TBI populations. The PTSD Checklist (PCL) is most commonly used to assess PTSD in veteran and military samples, followed by the Clinician-Administered PTSD Scale (CAPS). PTSD Checklist – Military version (PCL-M) and PTSD Checklist – Civilian version (PCL-C) were the most cited self-reported scales. DSM criteria were used to diagnose PTSD, while ICD was not cited in any of the studies we analyzed.

**Table 5:** **Characteristics of the studies and the scales and inventories used to assess PTSD after TBI**.

Reference	Study design	Study population	Number of analyzed studies/type of studies	Instruments
Betthauser et al. (108)	Systematic review	Military veterans with TBI	(30/47)	CAPS, DSM-IV, DSM-IV-TR, DTS NSI, PCL, PCL-C, PCL-M, PC-PTSD, TSI
Brady et al. (109)	Review	Veterans with PTSD, SUD, TBI	N/A	CAPS, IES-R, MPSS-SR, M-PTSD, NWS-PTSD, PCL-M, PSEI, SUD
Capehart and Bass (19)	Review	Veterans with TBI and PTSD	N/A	PCL
Carlson et al. (110)	Systematic review	TBI	31	BSI, CAPS, CIDI, IES, IES-R, PCL-M, PCL, PTSD-I, PDS, PSS, PSE, SCID
Carlson et al. (111)	Systematic review	mTBI/PTSD	34	CAPS, IES, PDS
Cooper et al. (31)	Systematic review	Military veterans with mTBI	(10/19)	CAPS, MPAI-4, NSI, PCL, PCL-C, PCL-M, PHQ
Daggett et al. (33)	Systematic review	Veterans/military with TBI	(1/17)	PCL-C, SCID
Garrelfs et al. (20)	Systematic review	ABI (TBI = 4)	(1/7)	SCID-I
Gill et al. (4)	Systematic review	TBI	28	CAPS, CIDI, IES, IES-R, SCID, PCL, PDS, PSS, PTSD-I
Harvey et al. (112)	Review	TBI	N/A	CAPS, CIDI, DIS, DSM-IV, ICD-10, IES, PSS, PSE, Penn Inventory, PTSD-I, SCID
Hesdorffer et al. (37)	Systematic review	TBI	(5/N/A)	CIDI, CAPS
Karr et al. (113)	Systematic review	Blast-related mTBI	9	CAPS
Kennedy et al. (114)	Review	Military veterans with mTBI or PCS	N/A	DSM-III, DSM-III-R, DSM-IV, DSM-IV-TR
Kim et al. (21)	Systematic review	TBI	(16/66)	CAPS, DSM-III-R, PSE, IES, CIDI, DSM-IV, PDS, PTSD-I, SCID
Matarazzo et al. (18)	Systematic review	Veterans/military with TBI	3	CAPS, PCL-S
Moore et al. (99)	Review	mTBI	N/A	DSM-III-R, MCMI-III, MMPI
O’Neil et al. (23)	Systematic review	Veterans/military with mTBI	(17/31)	CAPS, PCL, PCL-C, PCL-M, PCL-S, SCID
O’Neil et al. (22)	Systematic review	Veterans/military with mTBI	(17/31)	CAPS, PCL, PCL-C, PCL-M, PCL-S, SCID
McMillan et al. (115)	Review	TBI	N/A	DSM-III, DSM-III-R, DSM-IV, IES
Rice and Sher (116)	Review	Veterans with TBI	N/A	DSM, PCL, PHQ
Rogers and Read (25)	Review	TBI	(7/N/A)	BEC, IES, HSCL, PTSD-I, SCL-90-R, SCID
Soo and Tate (26)	Systematic review	mTBI	(1/3)	CAPS, IES
Steel et al. (45)	Systematic review	Traumatic injury	N/A	PTSD-I, CAPS, CIDI, SCID, SI-PTSD, DIS, PDS, IES-R, PC-PTSD, PCL, HTQ, M-PTSD, Civilian-MSS, Purdue PTSD, Penn Inventory, TSI
Tanev et al. (117)	Systematic review	TBI	N/A	ANAM, CAPS, DTS, NSI, PSS, PCL, SI-PTSD, TSI
Trachtman (118)	Review	Veterans with TBI	N/A	DSM-IV, Halstead-Reitan test, ICD-9, MMPI, PCL-M, PDHA, PDHRA
Wall (119)	Systematic review	TBI in military and veteran population	20	ANAM, BSI, DSM-IV, ICD-9, ICD-10PCL, M-PTSD, NSI, PCL-M, PCL-C, PDHA, PDHRA, PHQ

*PTSD, posttraumatic stress disorder; mTBI, mild traumatic brain injury*.

*Scales: ASDI, Acute Stress Disorder Interview; CAPS, Clinician-Administered PTSD Scale; CIDI, Composite International Diagnostic Interview; CSQ, Coping Style Questionnaire; Civilian-MSS, Civilian Mississippi Scale; DTS, Davidson Trauma Scale; DIS, Diagnostic Interview Schedule; M-PTSD, Mississippi Scale for Combat-Related PTSD; IES, Impact of Events Scale; IES-R, Impact of Events Scale-Revised; HTQ, Harvard Trauma Questionnaire; PCL, PTSD Checklist; PCL-C, PTSD Checklist – Civilian version; PCL-M, PTSD Checklist – Military version; PCL-S, PTSD Checklist – Stressor specific; PC-PTSD, Primary Care PTSD Screen; PDS, Posttraumatic Diagnostic Scale; PSE, Present State Examination; PSS, Posttraumatic Stress Scale; PSS-I, PTSD Symptoms Scale-Interview; SI-PTSD, Structured Interview for PTSD; PTSD-I, PTSD interview; SCID, Structured Clinical Interview for DSM; TSI, Trauma Symptoms Inventory*.

**Table 6:** **Scales and inventories used to assess posttraumatic stress disorder (PTSD) after TBI**.

Name of the scale	Abbreviation	Clinical utility
Acute Stress Disorder Interview (120)	ADIS	Interview schedule
Brief Symptoms Inventory (53)	BSI	Interview schedule
Civilian Mississippi Scale (121)	Civilian-MSS	Interview schedule
Clinician-Administered Posttraumatic Stress Disorder Scale (122)	CAPS	Interview Schedule
Composite International Diagnostic Interview (58)	CIDI	Interview schedule
Coping Style Questionnaire (123)	CSQ	Interview Schedule
Diagnostic and Statistical Manual 3rd edition [*DSM-III: n* (63)]	DSM-III	Diagnose
Diagnostic and Statistical Manual 3rd edition-revised [DSM-III-R (100)]	DSM-III-R	Diagnose
Diagnostic and Statistical Manual 4th edition [*DSM-IV* (64, 123)]	DSM-IV	Diagnose
Diagnostic and Statistical Manual 4th edition text revision [DSM-IV-TR (124)]	DSM-IV-TR	Diagnose
Diagnostic and Statistical Manual 5th edition [DSM-5 (125)]	DSM-5	Diagnose
Davidson Trauma Scale (126)	DTS	Interview schedule
Diagnostic Interview Schedule (83)	DIS	Interview schedule
Harvard Trauma Questionnaire (127)	HTQ	Interview schedule
Hopkins Symptom Checklist (69)	HSCL	Interview schedule
International Classification of Diseases 9th edition [*International Classification of Diseases (ICD)* (71)	ICD-9	Diagnose
International Classification of Diseases 10th edition (72)	ICD-10	Diagnose
Impact of Events Scale (101)	IES	Interview schedule
Impact of Events Scale-Revised (128)	IES-R	Interview schedule
Mayo-Portland Adaptability Inventory-4 (74, 129)	MPAI-4	Interview schedule
Mississippi Scale for Combat-Related Posttraumatic Stress Disorder (130)	M-PTSD	Interview schedule
Modified Posttraumatic Stress Disorder Symptom Scale (131)	MPSS-SR	Interview schedule
National Women’s Study Posttraumatic Stress Disorder module (132)	NWS-PTSD	Interview schedule
Patient Health Questionnaire (85)	PHQ	Interview schedule
Penn Inventory for Posttraumatic Stress Disorder (133)	Penn inventory	Interview schedule
Post-Deployment Health Assessment (134)	PDHA	Interview schedule
Post-Deployment Health Reassessment (135)	PDHRA	Interview schedule
Posttraumatic Diagnostic Scale (136)	PDS	Interview schedule
Posttraumatic Stress Scale (137)	PSS	Interview schedule
Potential Stressful Events Interview (138)	PSEI	Interview schedule
Present State Examination (87)	PSE	Interview schedule
Primary Care Posttraumatic Stress Disorder Screen (139)	PC-PTSD	Interview schedule
Posttraumatic Stress Disorder Checklist (140, 141)	PCL	Interview schedule
Posttraumatic Stress Disorder Checklist – Civilian version (142)	PCL-C	Interview schedule
Posttraumatic Stress Disorder Checklist – Military version (143)	PCL-M	Interview schedule
Posttraumatic Stress Disorder Checklist – Stressor specific (144)	PCL-S	Interview schedule
Posttraumatic Stress Disorder Interview (145)	PTSD-I	Interview schedule
Self-rating Posttraumatic Stress Disorder Inventory (146)	SIP	Interview schedule
Posttraumatic Stress Disorder Inventory Revised (147)	Revised PTSD inventory	Interview schedule
Posttraumatic Stress Disorder Symptoms Scale-Interview (137)	PSS-I	Interview schedule
Purdue Posttraumatic Stress Disorder (148)	Purdue PTSD	Interview schedule
Symptoms Checklist 90-Revised (106)	SCL-90-R	Interview schedule
Structured Clinical Interview for DSM (105)	SCID	Diagnose
Structured Interview for Posttraumatic Stress Disorder (149)	SI-PTSD	Interview schedule
Trauma Symptoms Inventory (150)	TSI	Interview schedule

## Discussion

### Depression

Major depression and dysthymia are frequently diagnosed using structured clinical interviews meeting DSM or ICD criteria. Depression often occurs in the first year after TBI (12). Estimates for posttraumatic depression range from 6 to 77% (151, 152), depending on diagnostic criteria, assessment methods, and timing post-trauma (22, 23, 152). Concomitant brain injury is a strong predictor of depression after TBI (13). In addition, poor mental health after TBI involves several factors, including young age at the time of injury, short duration between the injury and assessment, pain, lower levels of social support (153), and lack of hope (40). For those patients, consequences of depression include greater interpersonal difficulties, higher rates of unemployment (152), increased rates of distress, and problems with rehabilitation (154).

Some studies focus on specific TBI populations including the elderly, women, and veterans. Menzel (38) reviewed depression in the elderly after TBI, but the author found only one original study (104), leading to inconclusive findings. In their study, Levin et al. (104) assessed depressive symptoms by the Geriatric Depression Scale (GDS). Since this scale has been designed and standardized for geriatric populations with no history of TBI, there is a potential risk of overlapping the symptoms of TBI and depression. In addition, GDS cannot be used as a criterion for diagnostic assessment.

Seven studies addressed combat veterans with sustained TBI (18, 19, 22, 23, 31, 33, 36). They reported limited evidence that deficits and symptoms are distinct between veterans with or without a history of mild TBI (mTBI). O’Neil et al. (23) also highlighted a study (155) that showed an increased risk of suicide post-TBI compared to the non-TBI population. As we reported before, the BDI was the most cited inventory used in this sample. It contains 21 symptoms correlated with self-reported depression. The newer version of the BDI, the Beck Depression Inventory II (BDI-II), produced scores two points higher when compared to the oldest version for psychiatric outpatients (49). For this reason, comparisons between studies need to be carefully done.

One study addressed the literature focusing on women with TBI, comorbidity with depression, and hopelessness (40). The study analyzed symptoms both qualitatively and quantitatively. They concluded that mental health seems to deteriorate after TBI. Social isolation is of particular concern as a consequence of poor emotional functioning in these patients. In their study, the authors also reported on sex-based differences and limited data on the incidence of sex-specific depression.

Osborn et al. (17) showed the prevalence of major depression disorder (MDD) and dysthymia ranged from 14% using International Classification o Diseases (ICD-10) criteria to 43% using DSM-III criteria. For self-reported scales, the range of depression was between 16 and 33%. They found higher prevalence rates of depression using NFI than SCID-I, Schedules for Clinical Assessment in Neuropsychiatry (SCAN), or Mini-International Neuropsychiatric Interview (MINI). The occurrence of MDD and dysthymia appears to rise in the first 5 years after brain injury (from 21 to 43%). However, the majority of the studies used mixed TBI severity samples and did not report separate outcomes for these subgroups (17). Still, the HAM-D is wildly used to diagnose MDD in patients with TBI.

It is appropriate to use the standard diagnostic criteria for depression when evaluating persons with TBI. The CDE recommends scales and inventories to assess symptoms of depression in adults with TBI (15): the BDI-II, Brief Symptom Inventory-18 (BSI-18), CES-D, and Patient Health Questionnaire-9-Item (PHQ-9).

### Anxiety

Anxiety disorders are frequently comorbid after TBI; there is a complex and multifaceted relationship, considering that premorbid anxiety is a predictor of the development of depression and anxiety disorders post-TBI (156).

Anxiety disorders post-TBI have multiple etiologies, from environmental to biological/genetic. Anxious reactions usually follow brain injury occurring in the setting of traumatic events, such as motor vehicle accidents, falls, and assaults (157). We observed in our results that just one study aimed to analyze the sequelae of anxiety disorders after TBI (99). The other studies focused on the overall mental condition, including assessment of anxiety. One study analyzed the overall mental condition in a heterogeneous sample of patients with acquired brain injury (20). Three studies selected homogeneous samples: patients with closed TBI (17) and veterans/military personnel with mTBI (22, 23).

Osborn et al. (17) did a meta-analysis focusing on the prevalence of post-TBI generalized anxiety disorder (GAD). The results showed that approximately 11% of the patients were diagnosed with GAD after TBI, ranging from 2 to 28%. Taking into consideration the type of instrument used, the diagnostic scale ICD-10 was related to a lower prevalence rate (2%) of GAD after TBI, whereas the DSM-III-R was related to a higher prevalence (19%) of GAD (17). For interview schedules, SCAN showed a lower prevalence of GAD (2%), while the Schedule for Affective Disorders and Schizophrenia (SADS) showed a higher prevalence (28%) (17). The authors report differences in anxiety rates depending on the stages of TBI recovery. Thus, the timing of assessment may impact the number and severity of the symptoms, leading to bias in the results (158). The meta-analysis also showed a non-significant increase in the number of anxiety cases in the first 5 years post-trauma (17).

Two anxiety scales are suggested by the CDE for TBI populations: the Kiddie-Schedule for Affective Disorders and Schizophrenia (K-SADS) and Neuropsychiatric Rating Schedule (NRS). Interestingly, these scales were not extensively reported in this review, and the NRS was not reported in any of them.

The authors also recommend assessing substance abuse as a comorbidity of psychiatric conditions, especially in anxiety disorders (22, 24). The CDE suggests some questionnaires for this purpose: the Substance Abuse Questions from the TBI Model Systems Database, Alcohol Use Disorders Identification Test: self-reported version (AUDIT), and Alcohol, Smoking, and Substance Use Involvement Screening Test (ASSIST).

### Posttraumatic Stress Disorder

Posttraumatic stress disorder and GAD showed high prevalence after TBI and were both classified as anxiety disorders. In 2013, the DSM-5 classified PTSD as a trauma-stressor-related disorder, rather than an anxiety disorder. For this reason, we classified PTSD and anxiety disorders as separate psychiatric conditions.

In TBI patients, PTSD is usually related to a severe accident or injury, violent assault, domestic violence, war, or disaster (Criterion A – DSM-5). Prevalence rates of PTSD after TBI range from 3 to 59% (159, 160), while 43.9% of soldiers who reported loss of consciousness post-TBI met the criteria for PTSD (161). Our findings support previous results in which the heterogeneous range of diagnoses is due to the differences in assessment methods and methodologies of the original studies (111).

Depression, anxiety, and PTSD are usually comorbid conditions following TBI and may facilitate the persistence of its effects (162). Gill et al. (4) showed that psychological well-being is not predictive of the development of PTSD, but evidence suggests that individuals who have a history of psychological difficulties are at greater risk of developing PTSD after TBI.

Posttraumatic stress disorder is one of the most common mental health disorders affecting approximately 15% of veterans with no history of TBI. Nevertheless, the diagnosis rates of PTSD range from 33 to 65% in veterans with a history of TBI (163, 164). For this sample, the PCL is broadly used. The PCL-M and CAPS are the interview schedules most commonly used to assess PTSD in veterans and are recommended by the CDE (15). CAPS is considered the “gold standard” instrument for diagnosing and measuring the severity of PTSD, and it has been used with a variety of traumatized populations, including TBI (109, 165). There are different versions available, including CAPS to assess past-week, past-month, and lifetime symptoms. The PCL is a 17-item self-reported measure of PTSD symptoms and requires less time to complete than CAPS, which consists of a 30-item self-reported questionnaire. PCL is highly correlated with CAPS (*r* = 0.93), and it has favorable diagnostic efficiency (>0.70) and robust psychometric properties (165). For civilians with TBI, the PCL and CAPS are the most commonly used instruments to assess PTSD. However, the PCL-C and PTSD Checklist – Stressor specific (PCL-S) are preferable.

Overall, psychological variables, worsening general health, chronic pain, and somatic symptoms are associated with PTSD, especially in moderate to severe TBI. Comorbidities, such as PTSD and TBI, may unfavorably affect individuals more than suffering from any disorder alone (166). Some factors suggest how individuals with TBI might be more likely to develop PTSD. Somatic conditions and psychiatric disorders, such as PTSD, seem to perpetuate the illness condition in a loop (167). Those somatic conditions may present not only as risk factors but may also contribute to the persistence of other disorders, such as PTSD (4). This highlights the importance of therapy and rehabilitation for PTSD after TBI.

### Brain Function and Mental Health Post-TBI

Symptoms of anxiety, depression, and irritability often occur after TBI and affect mood centers, including the hippocampus, amygdala, and prefrontal regions of the brain (168). Psychological factors are potential contributors to poor recovery after mTBI (8). Since TBI etiologies are diverse, understanding the role of the neurobiological basis for behavioral dysfunctions can be complex. The neuroanatomical location of the head injury can play a role in the development of depression (45, 169). However, only a small portion of patients may sustain damage to the particular location and with a severity level necessary to produce a psychiatric syndrome while preserving adequate cognitive function; thus, a biological gradient can be very difficult to detect (25). Premorbid factors associated with psychiatric disorders are inconclusive. Family history of psychiatric disorders seems to be a predictor of depression (169) or PTSD in individuals who have experienced TBI (45). In addition, females have a higher risk of developing acute PTSD after motor vehicle accidents (170). However, some authors did not find this association in premorbid psychiatric illness with the development of PTSD (171).

Acquired brain lesions, especially those involving the prefrontal cortex may have a prominent role in developing and maintaining executive functions. These functions encompass a set of skills that allow for people’s adjustment and adaptation in the face of new situations and daily operation. Therefore, changes in executive functions are among the most common consequences resulting from TBI (172, 173). Depressive symptoms can also affect cognitive processes, inhibiting a patient’s ability to return to daily activities over the short-term. Prefrontal cortex disruption may result in impulsive behaviors and a higher risk of substance use disorders (174). Thus, there is evidence that TBI may increase the risk of drug or alcohol abuse in persons without a history of substance abuse before the injury, especially if the damage involves the orbitofrontal cortex (175). In our review, only short reports attempted to find associations between mental health and substance abuse. Hesdorffer et al. (37) reported that changes in drug and alcohol use usually preceded the occurrence of TBI, increasing the risk of head injury. One important point is that many of the studies analyzed in the reviews used current alcohol or substance abuse as exclusion criteria, possibly camouflaging their frequency of occurrence.

### Perspectives and Future Research

Considering the high incidence of TBI in the elderly (140–200 per 100,000 per year) and the relatively high prevalence of depression following TBI, it is reasonable to address the question of depression in the elderly following TBI (38). There is a gap in the literature addressing the evaluation and monitoring of elderly TBI patients.

One limitation related to veterans and military populations is that most of the analyzed data are from medical registries and clinical databases. It is necessary to have larger cohort studies, and it is also necessary to use standard methodology for the assessment of veterans and military populations.

Even in systematic reviews and meta-analyses, attrition bias may occur, as some studies showed that individuals who did not complete studies generally had more severe TBI (176–179) or, conversely, had less severe TBI (180). In addition, some studies did not control for confounding variables, such as the severity of trauma or the period post-TBI, reducing the generalizability of some results.

Many studies utilized self-report or semi-structured interviews for diagnosis. These results can lead to bias in the reported frequency of depressive disorder in this population. Self-report scales may not be reliable as patients with TBI may be unaware of their disabilities, and lack of awareness may lead to an underdiagnosis of psychiatric disorders (25). Another important variable relates to the recruitment of study participants. Outpatient and inpatient populations tend to vary in the intensity of their symptoms, particularly in the acute stage of trauma, when patients are often confused and disoriented. Many reviews analyzed mixed samples of trauma severity and different recruitment settings (17). Moreover, the majority of the instruments require that patients report their symptoms over the previous 2 weeks, and patients may have difficulty answering such questions or giving reliable responses, particularly in the acute stages of trauma.

Another important issue is the analysis of premorbid psychiatric conditions, a factor that may bias results. Finally, as we are reporting on specific information from selected reviews in the field, there is the risk of publication bias.

## Conclusion

There is significant variability in the types of assessments used in the evaluation of psychiatric disorders after TBI, and consequently, there is also variability in the reported prevalence of such disorders. We analyzed meta-analyses and systematic reviews focusing on the most prevalent psychiatric conditions, and we observed a heterogeneous pattern related to their assessment and diagnosis in TBI populations. Depression after TBI is a well-established condition with homogeneous studies. Anxiety and PTSD disorders have been studied in a heterogeneous way, usually comorbid with other psychiatric disorders. The variability of clinical findings raises the importance of the instruments used to assess these patients. Finally, some scales and inventories designed for the general community may not be appropriate for patients with TBI (152).

## Author Contributions

AZ – had the idea of the review, organized the search method, and wrote the manuscript. JV – helped to write the manuscript and did the tables. FF – reviewed the manuscript and helped the elaboration of the manuscript. PR – helped to write the manuscript. CB – helped on the tables review. ML – helped to review the manuscript. WP – reviewed the manuscript.

## Conflict of Interest Statement

The authors declare that the research was conducted in the absence of any commercial or financial relationships that could be construed as a potential conflict of interest.
